# Intramuscular stimulation vs sham needling for the treatment of chronic midportion Achilles tendinopathy: A randomized controlled clinical trial

**DOI:** 10.1371/journal.pone.0238579

**Published:** 2020-09-08

**Authors:** Lyndal Solomons, Jenny J. Y. Lee, Margaret Bruce, Lynita D. White, Alex Scott

**Affiliations:** 1 Department of Physical Therapy, University of British Columbia, Vancouver, Canada; 2 Allan McGavin Sports Medicine Centre, Vancouver, Canada; 3 Centre for Hip Health and Mobility, Vancouver Coastal Health Research Institute, Vancouver, Canada; 4 Kinetic Rehabilitation Centre, North Vancouver, Canada; 5 Tall Tree Physiotherapy & Health Centre, Vancouver, Canada; Kanazawa University, JAPAN

## Abstract

**Background:**

The insertion of filiform needles intramuscularly (a.k.a. intramuscular stimulation/dry needling) has been suggested as a possible treatment for various painful musculoskeletal conditions. Our aim was to answer the question, is intramuscular stimulation more effective than sham intramuscular stimulation/dry needling for the treatment of Achilles tendinopathy?

**Methods:**

52 participants with persistent midportion Achilles tendinopathy began and 46 completed one of three treatment protocols which were randomly assigned: (G3) a 12-week rehabilitation program of progressive tendon loading plus intramuscular stimulation (n = 25), (G2) the same rehabilitation program but with sham intramuscular stimulation (n = 19), or (G1) a reference group of rehabilitation program alone (as an additional control) (n = 8). The *a priori* primary outcome measure was change in VISA-A score at 12 weeks–VISA-A was also measured at 6 weeks, and at 6 and 12 months. Secondary outcome measures include the proportion of patients who rated themselves as much or very much improved (%), dorsiflexion range of motion (degrees), and tendon thickness (mm).

**Results:**

The study retention was 94% at 12 weeks and 88% at 1 year. VISA-A score improved in all three groups over time (p<0.0001), with no significant difference among the three groups in VISA-A score at the start of the study (mean ± SD: G3 59 ± 13, G2 57 ± 17, G1 56 ± 22), at 12 weeks (G3 76 ± 14, G2 76 ± 15, G1 82 ± 11) or at any other timepoint. The percentage of patients who rated themselves as much or very much improved (i.e. treatment success) was not different after 12 weeks (G3 70%, G2 89%, G1 86% p = 0.94), or at 26 (p = 0.62) or 52 weeks (p = 0.71). No clinically significant effects of intervention group were observed in any of the secondary outcome measures.

**Conclusion:**

The addition of intramuscular stimulation to standard rehabilitation for Achilles tendinopathy did not result in any improvement over the expected clinical benefit achieved with exercise-based rehabilitation alone.

## Introduction

Mid-portion Achilles tendinopathy (AT) is accompanied by pain and impaired function in the Achilles tendon [1]. It is typically accompanied by structural changes visible on ultrasound such as thickening of the tendon, a feature which is present early in the development of pathology [2]. The pain of Achilles tendinopathy may be accompanied by altered central nervous system processing, such as reduced conditioned pain modulation and mechanical secondary hyperalgesia [3, 4] although others have argued that Achilles tendinopathy pain is primarily driven by peripheral nociception [5] perhaps as a result of local nociceptive substances [2, 6].

Achilles tendinopathy is common in runners, with a recent cohort study reporting a 24% incidence among runners [7]; in that study, years of activity and weekly mileage were identified as risk factors, although not all studies have found such associations [8]. In another study, the cumulative incidence of Achilles tendinopathy was 24% in former elite athletes from various sports (running, athletics, soccer, hockey, etc) compared to 5.9% in matched controls [9]. The prognosis of Achilles tendinopathy is variable; in club-level adult and youth athletes, the median time to return to play from Achilles tendinopathy was 1.7–12.5 days, with an upper range of 30 days [10]. Others have reported that ongoing symptoms are present 5 years later in over a third of recreationally active adults who enrolled in a clinical trial [11].

Current recommended treatment for those with persistent Achilles tendinopathy emphasizes tendon-loading exercises and activity modification/education [1, 12] with eventual referral to surgery for severe long-standing cases [13, 14]. Placebo-controlled studies of proposed adjunct treatments such as shockwave therapy [15–17] or topical glyceryl trinitrate [18, 19] have not clearly demonstrated a benefit for Achilles tendinopathy, showing small improvements [16], improvements reported by a single study [19], or no apparent benefit [15, 17, 18]. There are very few placebo-controlled studies of injection therapies [20–26] or analgesics (either topical [27] or oral [28]) to inform clinical decision-making.

The insertion of filiform needles (FNs) intramuscularly, a.k.a. intramuscular stimulation (IMS), has been suggested as a possible treatment for Achilles tendinopathy [29]. Gunn et al [30] suggested that, in people with Achilles tendinopathy, the gastrocnemius and soleus muscles may become “shortened” (i.e demonstrate reduced extensibility) and deliver excessive mechanical stress to the Achilles tendon. They suggested that this muscle shortening may result from the formation of taut bands (sustained contraction in localised sections of the muscles), and that the insertion of FNs into these taut bands can assist in relaxing them, restoring their extensibility and thereby normal mechanical loading of the tendon. They also suggested that local gastrocnemius and soleus muscle banding/shortening may often be accompanied by muscle banding/shortening in other muscles that share the same segmental nerve supply as the gastrocnemius, soleus and Achilles tendon, i.e. L5-S2. They hypothesised that the reason for this may be neuropathic dysfunction in the affected segments [31].

There is great variability in the way FNs are used to treat musculoskeletal pain both in practice and in research settings, and there is no research on the use of FNs intramuscularly to treat Achilles tendinopathy. For other conditions, such as chronic low back pain, it has been suggested that dry needling may be a useful adjunct treatment, however further high quality studies are needed [32]. There is only one published study that uses a needle insertion approach to treat Achilles tendinopathy, conducted by Zhang et al [33], but it is unclear where the FNs were inserted (skin, muscle, tendon etc) and the conclusion of the study (that the effect of acupuncture-style needling on pain and function was superior to exercise) may be questioned. In their 2016 systematic review Cox et al [34] rated the study by Zhang et al as having a low risk of bias, however the acupuncture group (1) was not blinded, (2) was not compared to a sham group, and (3) received 24 visits over 8 weeks compared to apparently a single visit in the exercise group, which achieved lower-than-expected outcomes for exercise-based treatment [33]. Thus, there is very minimal evidence on which to base a recommendation for the use of dry needling of any kind in people with chronic Achilles tendinopathy.

The primary purpose of this study was to compare the clinical status of people with Achilles tendinopathy who receive IMS (G3) to those who receive sham needling (G2); a reference group (G1) received no needling. To our knowledge, this is the first placebo/sham controlled randomized controlled trial of an intramuscular dry needling technique for Achilles tendinopathy.

## Methods

### Study design

This was a parallel, randomized, single-blind, controlled trial at a single site: the University of British Columbia (Vancouver, Canada). A single investigator (LS) provided all treatments. Three treatment and data collection locations were used: the Centre for Hip and Health Mobility at Vancouver Coastal Health Research Institute (Vancouver), Kinetic Rehabilitation Centre (North Vancouver), and Canopy Integrated Health (North Vancouver). Patients were recruited by placing advertisements in public locations, local newspapers, and a Facebook page, and an entry in the Vancouver Coastal Health Research Institute trial database and newsletter. All patients provided written informed consent. The study was approved by the UBC Clinical Research Ethics Board (H12-02008). The trial was registered and kept up to date at ISCRTN.com (70177540). As described below in the section on enrolment, the study took place in two phases but with identical outcome measures such that the data set could be combined and analyzed as a whole.

### Participants

Inclusion criteria were; 19 to 60 years of age, fluent in English, minimum symptom duration of 3 months, evidence of midportion Achilles tendinopathy on physical examination, i.e. pain location isolated to the mid-portion of the Achilles tendon and progressive loading causing increasing pain (double leg toe-raise, single leg toe-raise, jump, hop, hop for height, hop for distance), and indications by Gunn IMS assessment of neuropathic change in the L5-S2 segmental levels including the presence of taut muscle bands amenable to IMS. Exclusion criteria were; IMS contraindications (infection in the area, pregnancy, bleeding disorders, history of bacterial endocarditis, post-surgical implant in the past four to six months, major surgery in the last three months), previous treatment with intramuscular dry needling (for blinding purposes), true leg length difference of greater than ½”, systemic inflammatory disease, previous corticosteroid injections, recent fluoroquinolone use, and the presence of other syndromes that cause pain in and around the Achilles tendon (determined by history-taking and physical examination).

Age, gender, symptom duration and physical activity level (International Physical Activity Questionnaire) were recorded at visit 1.

### Enrolment and randomization

After informed consent was obtained, and before any clinical assessment or baseline data collection, participants were randomized by a study coordinator who had no role in the initial assessment or treatment. The study coordinator was provided with a simple (unblocked, unbalanced) random allocation sequence with 42 allocations into three groups, generated by the primary investigator (AS) using a random sequence generator (Microsoft Excel). This list was used to allocate the first 21 participants (recruited from April 2013 to April 2014) into G3, G2 or G1 conditions. In summer of 2014, an amendment to the protocol was submitted to the funder, ethics board and trial registry such that further allocations would be only to the G3 and G2 groups to focus on the primary question—comparison between IMS and sham needling. Thereafter a revised (unblocked, unbalanced) random allocation sequence with a further 42 allocations was generated. The remaining participants were recruited and allocated to G3 or G2 from October 2014 –December 2018. The same research assistant assigned participants to groups sequentially as they enrolled using the random allocation sequence and notified the researcher administering the interventions (LS) of participants group allocation just prior to their first treatment session. Only AS and a research assistant not involved in participant intervention or assessments had access to the random allocation sequence.

### Blinding

Participants assigned to a needling group were blinded to their group allocation (IMS or sham IMS) until after having received their nine treatments and completed their 12-week outcome measures. It was not possible to blind the treating therapist (LS) to participant group allocation. Those assessing ankle range measures were blinded to group allocation. Ultrasound scans were taken by LS and interpretation of ultrasounds was performed by a research assistant blinded to group allocation. After 12 weeks, participants who received needling were asked which group they thought they were assigned to, and their answer was categorized as correct or incorrect.

### Important changes to methods after trial commencement (such as eligibility criteria), with reasons

Initially, prior experience with Traditional Chinese Medicine Acupuncture was an exclusion criterion. This was removed prior to the start of recruitment (in April 2013) as it was seen as potentially an undue hindrance to recruitment. After enrolment of the first 21 participants, we amended the allocation ratio (as described above) to focus on the primary question (difference in outcome between IMS (G3) and sham IMS (G2) groups by making no further allocations to the exercise-only reference group (G1).

### Interventions

All participants (G3, G2, G1) received a standardised 12-week physiotherapy program provided by one of the investigators (LS), including a progressive isometric, concentric and eccentric training and kinetic chain strengthening program. All groups were prescribed the same standardised exercise programme designed to initially maintain (as pain allowed) and then, as tolerated, increase the ability of the muscle-tendon unit and kinetic chain to absorb load [1]. The programme was progressive, and eccentric loading of gastrocnemius-soleus-Achilles tendon complex loading was incorporated throughout. The first phase focused on isometric loading, the second on concentric and then load, range and speed were gradually increased [5]. Participants were progressed based on a “twenty-four-hour response” to exercise, specifically, there was not to be increased pain or stiffness the morning after undertaking the exercise, however pain during exercise was allowed. Participants were advised to keep the discomfort during activities of daily living to a level of no more than 5/10 on a scale of 0 to 10 where 0 is no pain at all and 10 is the worst possible imaginable pain. Participants filled out a training diary–the number of sessions completed was calculated as a percentage of prescribed sessions.

G3—The IMS treatment group received intramuscular dry needling treatment (APS Dry Needling Needles by Agupunt; Barcelona, Spain; 0.25–0.30 x 25-75mm) once a week for the first six weeks of the trial and once every two weeks for the remainder, resulting in nine treatments overall. The choice of needle insertion points was individualised for each participant based on their assessment findings and using the neuropathic model approach described by Dr. C. Chan Gunn [31]. This approach considers the potential contribution of a dysfunctional nervous system to myofascial pain syndromes, and in the case of Achilles tendinopathy with a particular focus on treating dysfunction in the L5-S2 segments. Treatment involved the insertion of FNs into taut muscle bands (as palpated by the treating therapist) in both the spinal region and the lower limbs.

G2—The sham IMS group, received eight FNs (APS Dry Needling Needles by Agupunt; Barcelona, Spain; 0.25 x 25mm) that were inserted superficially (1–2 mm maximum depth) in the buttock, posterior thigh and calf regions and were left in situ for ten minutes. The schedule and number of visits was the same as for G3. To avoid mimicking either IMS or purported TCMA effects: needles were inserted away from any taut muscle bands that were palpated by LS and were inserted superficially so that no muscle penetration occurred; purported TCMA meridians in the treatment area and TCMA points used to treat heel pain specifically were avoided; and it was ensured that no “deqi” sensation was elicited (i.e. aching, soreness, pressure) [35].

G1 –This reference group (actively allocated only during the first portion of the study period, as described above) only received the exercise program. This intervention group was included as an additional, internal reference primarily to ensure that the exercise program was performing as expected.

### Outcome measures

The outcome measures were designed and registered prior to the publication of a recent consensus on outcome measures [36]. There were no changes to outcome measures after registration of the protocol at ISCRTN, although we did alter the ultrasound analysis from what was described in our ethics protocol by focusing on tendon thickness rather than echo texture. The primary outcome for which the study was powered was the change from baseline in 12 week VISA-A (Victorian Institute of Sports Assessment–Achilles), a valid and reliable disease-specific outcome measure [37] which also includes an activity-related pain scale. The VISA-A score was also measured at 6, 26 and 52 weeks.

Other secondary outcome measures were included *a priori*:

(1) A written, 7-point, patient-rated Likert scale of Global Rating of Change (GROC) was filled in by patients, from 1 (very much improved) to 7 (very much worse). Ratings of “very much improved” or “much improved” were considered as treatment success (6, 12, 26, 52 weeks).

(2) Dorsiflexion range of motion was measured in two weightbearing positions (knee bent or straight) using an inclinometer placed midway along the anterior surface of the tibia (weeks 0, 12 and 52) [38]. Prior to measurement, participants performed stretches in a standing lunge with the middle of the heel and the second toe aligned in a straight line, with both the back knee straight and the back knee bent (15 second stretch, five times each). Range of motion was measured by one of two physiotherapy assessors who were both blind to treatment allocation.

(3) Tendon thickness in the antero-posterior plane at the point of maximal tendon thickening was measured using grey-scale B-mode ultrasound images (weeks 0, 6, 12, 26 and 52). Participants lay prone on an examination bed with their foot positioned and stabilised by the person taking the scan such that the tendon was “flat” (i.e. lay passively straight) to achieve a perpendicular alignment between the Achilles tendon and the UTC transducer (Smartprobe 10L5; Terason 2000, Teratech, USA; UTC Technologies, Oldemarkt, Netherlands), i.e. in an individual-specific degree of dorsiflexion. The transducer (in its tracker) was positioned and stabilised by the person taking the scan so that the centre of the ultrasound head was placed over the centre of the posteromedial aspect of the tendon (the region most commonly affected by Achilles tendinopathy) to allow the ultrasound beam to interface with this region at a perpendicular angle. Once the foot and transducer were fixed in position, adjacent transverse images (2D) of the AT were automatically captured at every 0.2 mm for 12 cm along the tendon axis and compiled to create a 3D scan for tomographic visualization in the transverse, sagittal, and coronal planes. Each subsequent scan was obtained using the same method. The ultrasound probe was a Smartprobe 10L5 (Terason 2000, Teratech, USA) attached to an Ultrasound Tissue Characterization tracking system (UTC Technologies, Oldemarkt, Netherlands). Transverse images (2D) of the AT were automatically captured every 0.2 mm for 12 cm along the tendon axis and compiled to create a 3D scan for tomographic visualization in the transverse, sagittal, and coronal planes. Scans were de‐identified and analyzed with blinding of the time point and participant ID. To analyze the scans, the site of maximal thickening was identified at week 0. The same location was used for all subsequent measures by measuring caudally from the calcaneal insertion.

### Sample size calculation

We assumed that the change in VISA-A from 0 to 12 weeks with exercise alone would be 20 points [37], with a normal distribution and a standard deviation of 12 [23]. We assumed the minimum clinically important difference in VISA-A score to be 12 points [37]. Given these assumptions, to answer the primary question, the trial was powered (at 0.80, with alpha of 0.05) to detect a difference of 12 points between the G3 (IMS) and G2 (sham needling) groups, which for an independent t-test would require a sample size of 16 in each group.

### Statistical analysis and treatment of missing data

Statistical analysis was conducted in R 3.6.0. Data are presented to 2 significant figures with the standard deviation (SD) in parentheses. For the primary and secondary outcome measures, an independent blinded statistical analysis was conducted by a professional statistician. For the main outcome measure (VISA-A), linear modeling was employed to incorporate the repeated nature of the measurements. For the primary endpoint of VISA-A, we used a model of VISAtime = αSUBJECT+VISAbaseline∗group+log(time)+ϵtime. The covariance structure was Correlation(ϵt1,ϵt2) = ϕ|t1−t2|, to account for the fact that the longer the time interval between two observations, the weaker their correlation is. We also tested for interactions between group and time, and between baseline VISA and group. The same modelling process and covariance structure was used for dorsiflexion range of motion (both bent and straight knee) and tendon thickness. GROC was treated as a binary variable (success or failure) and tested using Pearson’s chi-squared test. All available data for all patients who received their allocated treatment were included in the analysis, regardless of whether their data set was complete. All patients were analyzed according to their allocated treatment (i.e. intention to treat). We did not impute or replace any missing values but rather fitted all the available data to the model. Actual, not modelled, data are presented. An interim analysis was planned at the midpoint of recruitment, and this was conducted slightly ahead of schedule after the first 18 subjects were enrolled, in order to support release of further grant funds.

## Results

### Participants

**Fig 1:**
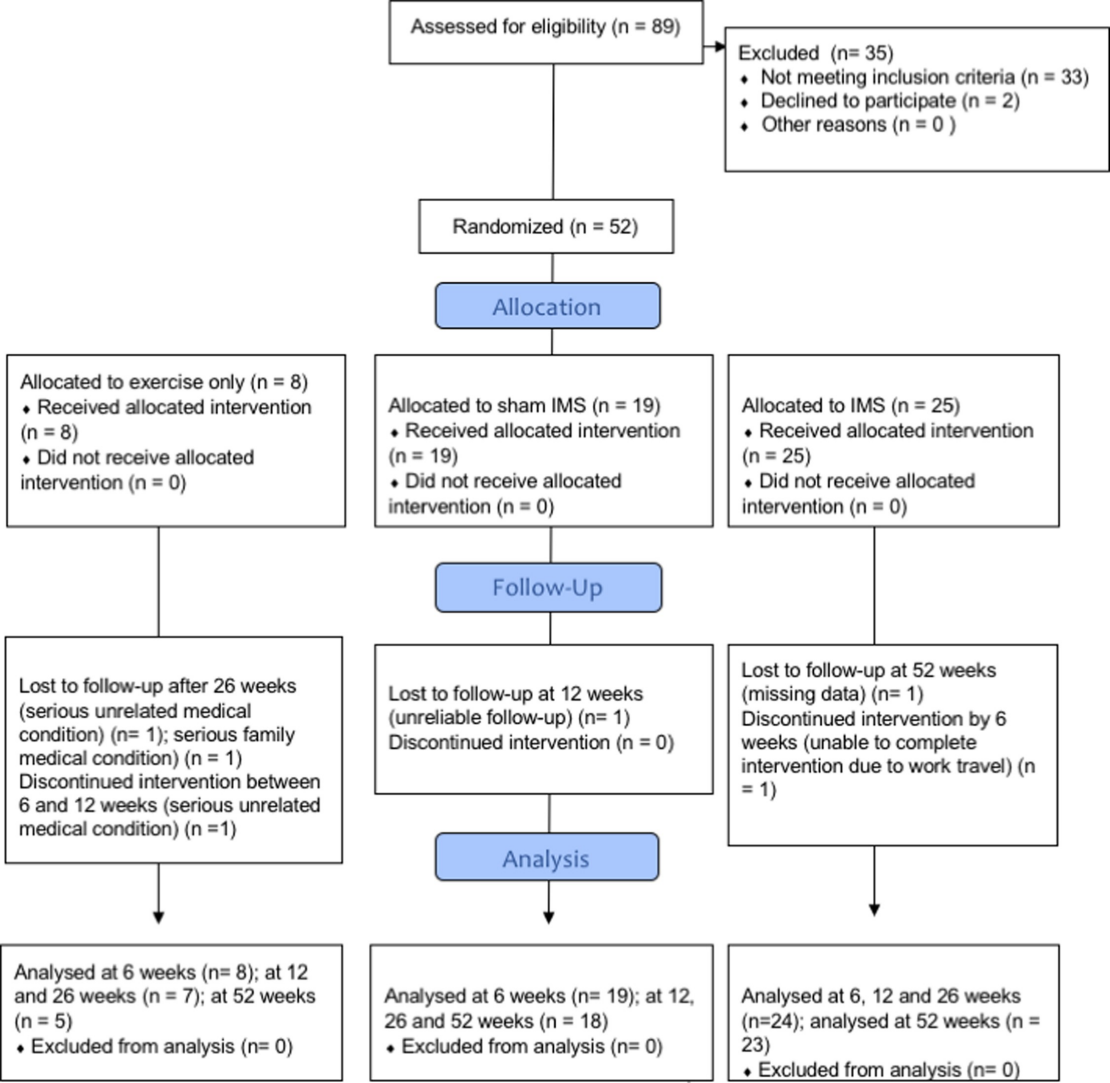
Consort diagram.

Recruitment began in April 2013 and was completed in December 2018. The trial was stopped because the target sample size for G2 and G3 were achieved. Fifty-two participants were allocated into 3 groups (Fig 1) which were demographically and clinically similar (Table 1). Thirty-one (60%) participants identified running as the likely mechanism of injury to their Achilles tendon. Other reported mechanisms of injury were walking (7, 13%), jumping (3, 6%), soccer (3, 6%), hiking (2, 4%), basketball (1, 2%), bus driving (1, 2%), swing dancing (1, 2%), squash (1, 2%) and tennis (1, 2%). Twenty participants (64% of runners) had stopped running due to their Achilles pain. On entry to the study, 24 participants were walking for exercise, 2 were hiking, 13 were running, 1 per activity were still participating in soccer, basketball, bus driving and swing dancing.

**Table 1:** Baseline characteristics of study subjects.

Baseline Data	Exercise only (Group 1)	Sham IMS (Group 2)	IMS (Group 3)	Overall
Female, male	5 (63%), 3 (38%)	8 (42%), 11 (58%)	15 (60%), 10 (40%)	28 (54%), 24 (46%)
VISA-A: mean (SD)	56.1 (24.1)	58.5 (17.0)	57.7 (12.7)	57.8 (15.9)
Age: mean (SD)	47 (7.2)	46 (7.6)	51 (5.8)	48 (7.0)
Physical Activity level (IPAQ)	0 (0%)	0 (0%)	1 (42%)	1 (2.0%)
1 = inactive, 2 = minimally active, 3 = highly active	0 (0%)	6 (67%)	9 (38%)	15 (30%)
	8 (100%)	3 (33%)	14* (58%)	35 (67%)
Symptom Duration: mean (SD) in months	7.6 (7.5)	18 (16)	21 (15)	18 (15)

*One missing data point.

### Success of blinding

At 12 weeks, when asked to state what group they thought they were in, most participants had difficulty selecting one or the other group. There were three missing data points; two from G3 and one from G2. In G3, 81% of participants correctly guessed they received IMS. This is perhaps understandable because IMS typically elicits noticeable sensations. In G2, 67% of participants correctly guessed they were in the sham needling group. Thus, the blinding may be said to be only partially successful.

### Missing data

Most missing data were a result of participants’ leaving the study (ie, all subsequent measurements after a certain time point were missing), yielding a retention rate of 94% at 12 weeks and 88% at 1 year. After 1 year, the number of participants who had left the study were 2 (G3), 1 (G2), and 3 (G1). Missing tendon thickness values also occurred at a particular time point for 3 participants due to unusable or missing ultrasound scans: 1 at 6 weeks and 2 at 26 weeks. GROC values were missing for a G3 participant at 26 weeks, but not at the other timepoints. The linear modeling did not impute missing values but fitted all available data to the model. All analysis was completed by the original assigned group.

### Exercise compliance

Based on the information recorded by patients in the exercise diaries, the percentage of participants who completed at least 75% of their prescribed exercise sessions was 83% (G3), 92% (G2) and 100% (G1).

### Primary outcome: VISA-A at 12 weeks

**Fig 2:**
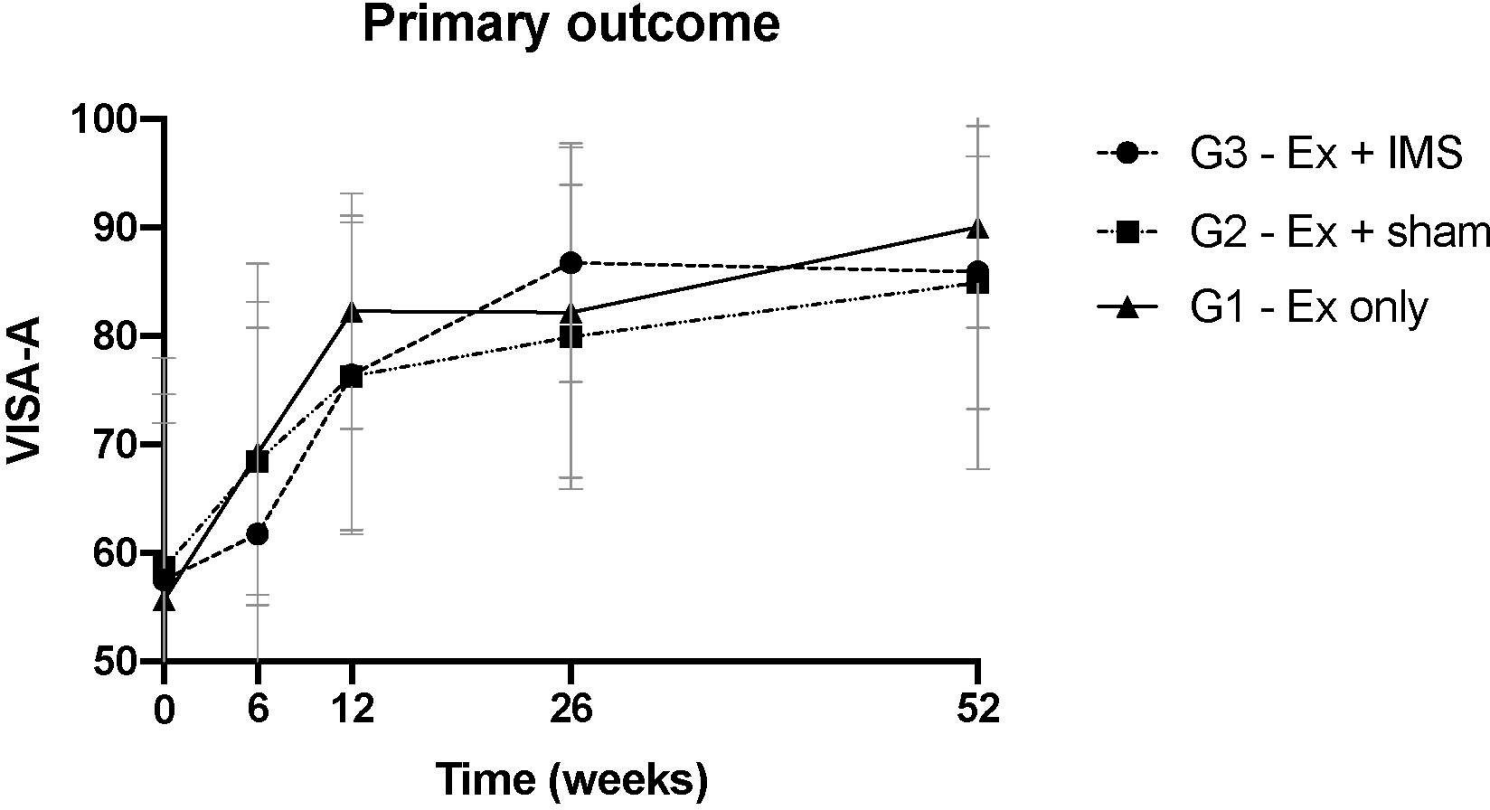
Change in symptom severity over time. Mean and standard deviation are shown. VISA-A: Victorian Institute of Sport Assessment–Achilles. Ex–exercise. IMS–intramuscular stimulation (i.e. dry needling).

Across all groups, the majority (71%) of patients experienced an improvement in VISA-A score of ≥ 12 points from 0 to 12 weeks; the improvement in VISA-A in all groups was statistically significant (p<0.001). There was no significant difference in the magnitude of improvement between treatments (p = 0.13), and no significant interaction between group and time (p = 0.51) or group and baseline VISA-A (p = 0.32) (Fig 2). The mean improvements in VISA-A score were: G3, 18 (13); G2, 18 (11); G1, 26 (21).

### Secondary outcomes

**Fig 3:**
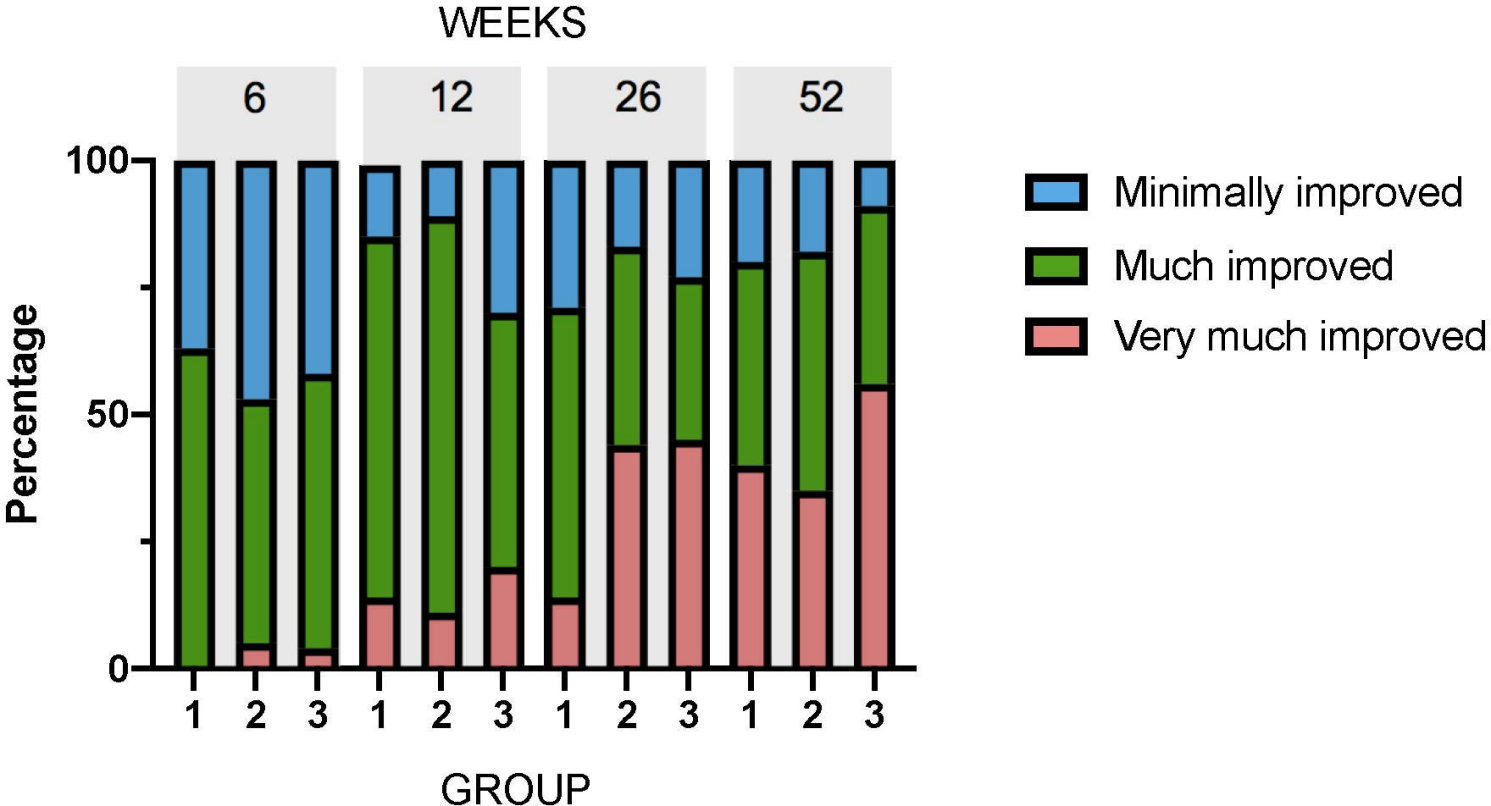
Comparison of global rating of change (GROC) over time. 1 = very much improved, 2 = much improved, 3 = minimally improved. Group 1—exercise only, Group 2—sham IMS, Group 3—IMS. Not shown on graph: one participant in Group 3 was unchanged (GROC = 4) at 26 weeks, and one participant in Group 4 was minimally worse (GROC = 5) at 52 weeks.

The percentage of patients who rated themselves as much or very much improved (i.e. “treatment success”) was not significantly different at any timepoint (Table 2: 12 weeks, p = 0.94; 26 weeks, p = 0.62; 52 weeks, p = 0.71.). Pearson’s Chi-squared testing showed there was no significant difference in the distribution of GROC rating between Groups 1, 2 and 3 at any time point (Fig 3).

**Table 2:** Secondary outcome measures of patients treated for Achilles tendinopathy^a^.

	Baseline	6 weeks	12 weeks	26 weeks	52 weeks
**Tendon thickness (mm)**					
**G3**	9.2 (2.3)	9.3 (2.1)	8.9 (2.1)	9.0 (2.3)	8.6 (2.4)
**G2**	8.2 (1.6)	8.2 (1.7)	8.1 (1.6)	7.9 (1.6)	7.5 (1.3)
**G1**	8.7 (1.7)	8.1 (2.0)	8.4 (2.6)	8.2 (2.6)	6.3 (0.3)
**Range of motion, SK (degrees)**					
**G3**	41 (11)	─	45 (11)	─	47 (11)
**G2**	44 (8.2)		43 (7.6)		43 (9.5)
**G1**	34 (4.8)		37 (1.8)		36 (9.3)
**Range of motion, BK (degrees)**					
**G3**	45 (8.6)	─	48 (9.0)	─	50 (7.8)
**G2**	47 (7.9)		47 (7.3)		46 (10)
**G1**	42 (4.6)		43 (2.4)		45 (4.7)
**Treatment success (%)**					
**G3**	─	58	71	78	92
**G2**		52	89	83	82
**G1**		63	85	71	80

^a^ G3, Intramuscular dry needling + exercise; G2, Sham needling + exercise; G1 Exercise only. Values are presented as mean (SD). SK, Straight knee. BK, bent knee.

On the whole, the tendon thickness significantly decreased over 12 months regardless of group allocation (p<0.01) (Table 2), despite that fact that there was a significant interaction between baseline value and group (p = 0.007) indicating a slight allocation bias for this outcome measure. Dorsiflexion range of motion did not differ between groups when measured either with the knee straight (p = 0.124) or bent (p = 0.474), and did not significantly improve over time (p = 0.277 and p = 0.328 and respectively); baseline range of motion was the only significant predictor (p<0.001 for both straight and bent knee).

### Side effects

Most individuals in the IMS group reported acute localized sensation when the needles were inserted to the depth of the muscle, reporting a deep ache of variable intensity and/or a muscle twitch/contraction. Many in the IMS group also reported post-treatment soreness of variable intensity and duration. Both of these were expected treatment effects, and likely contributed to the difficulty in blinding to group allocation, as in contrast there was almost no sensation reported from needle insertion to the depth of the skin only in the sham IMS group–only the very occasional reporting of mild sharpness—and no reporting of post-treatment soreness. When a sensation of sharpness on needle insertion occurred in either needling group, the needle was immediately removed, and replaced at an adjacent site where no sharpness was elicited and left in situ in that location. As expected, occasional minor bruising resulted from some of the needle insertions in the IMS group. There were no severe adverse events reported (including pneumothorax, organ injury, nervous system injury or infection). No patient withdrew due to needling side effects.

## Discussion

This study failed to demonstrate a greater improvement in symptoms of Achilles tendinopathy in patients who received IMS (intramuscular dry needling, using filiform needles) and exercise, compared either to sham IMS and exercise, or to exercise alone. The magnitude of improvement observed in all three groups in the primary outcome measure (VISA-A) was as expected, based on previous studies with similar exercise programs [39].

In some jurisdictions, intramuscular dry needling, including IMS, is widely used by physiotherapists and other health professionals as an adjunct treatment for tendinopathies and other conditions, with some support for conditions such as low back pain [32]. There is minimal evidence on whether this treatment technique (or other needle-based techniques such as acupuncture) is effective for Achilles tendinopathy, or other tendinopathies.

A variety of theories have been advanced about the potential mechanisms whereby intramuscular dry needling may influence musculoskeletal pain; however, since there did not appear to be an important clinical effect on Achilles tendon pain in this trial, we will not speculate about potential treatment mechanisms here. Regarding the possible effect in this study of IMS on muscle length, however, the main mechanism that has been proposed for this effect is the induction of a local twitch response (LTR). The LTR has been described as a reflex contraction of the muscle fibres that form a taut band that can be elicited by inserting a needle into the band [40]. LTRs induced by intramuscular dry needling have been associated with the following changes within muscles: a reduction in excess electrical activity [41], a reduction in local excess levels of the neurotransmitters Substance P and calcitonin gene related peptide [42], and a reduction in mechanical hypersensitivity [43]. We did not document whether an LTR occurred with intramuscular needle insertions in the IMS group in this study.

Another limitation is that we were not able to objectively measure one of the prescription parameters for the type of needling used here (presence of taut bands within muscles of the L5-S1 segment). We recognise that a technology has not been developed that is able to identify or measure differences in hardness of different regions of muscle with the robustness required for a study of this kind, although shear wave elastography shows promise if the technology can be further refined. Nevertheless, manual therapists attest to the ability of the human sense of touch to palpate differences in hardness of different regions of the same muscle. This was the method used to determine the location for intramuscular needle insertions in this study, and we acknowledge this method as a limitation.

This study has some other limitations. Our sample size was relatively small, particularly for the exercise-only group which was mainly included as a reference group to ensure that the exercise program was performing similar to the reports. Our power calculation assumed that the standard deviation of the VISA-A score would be 12, and in fact it was 14. This indicates that for the primary outcome, the required sample size to detect a difference between sham and IMS needling (the main study question) was 22 per group, which was nearly met for G2 and G3. Thus, although we cannot exclude the possibility that the trial was under-powered, nevertheless it seems unlikely that including a few more participants in the sham IMS group would alter the observed mean improvement in VISA-A (G3, 18 (13); G2, 18(11)). An interesting finding is that blinding was only partially successful, and yet despite this a placebo effect was not observed for either needling group. The results of this study cannot be generalized to other patient populations such as elite athletes or those with insertional Achilles tendinopathy, or to patients who may be treated with needling alone rather than in combination with exercise.

## Conclusion

In conclusion, the addition of intramuscular stimulation to standard rehabilitation for Achilles tendinopathy did not result in any improvement over the expected clinical benefit achieved with sham needling and exercise-based rehabilitation.

## Supporting information

S1 File Study protocol. (DOCX)

S2 File CONSORT 2010 checklist. (DOC)

S1 Table Minimal data set. (XLSX)
