# Supplementary material for: Intramuscular stimulation vs sham needling for the treatment of chronic midportion Achilles tendinopathy: A randomized controlled clinical trial
Source: PLoS One. 2020 Sep 8;15(9):e0238579. doi: 10.1371/journal.pone.0238579 (PMC7478532; doi:10.1371/journal.pone.0238579)
Supplement: S1 File — (DOCX) [file pone.0238579.s001.docx]

# Intramuscular stimulation and eccentric exercise vs eccentric exercise plus sham needling or eccentric exercise alone for the treatment of chronic midportion Achilles tendinopathy: A randomized controlled clinical trial pilot study

**Principal Investigator:** Scott, Alexander RPT, PhD

UBC Department of Physical Therapy

772-2635 Laurel St, Vancouver, BC Canada V5Z 1M9

Tel: 604 875 4111 x21810

**Co-Investigator(s):**

Lyndal Solomons RPT, FCAMT

Kinetic Rehabilitation Centre

151 15th Street East

North Vancouver, BC V7L 2P7

Tel: 604 987 7644

# Background – Review of evidence-based treatment for chronic midportion Achilles tendinopathy

Achilles tendinopathy (AT) is a common condition, particularly in those who run. It is a chronic, activity-limiting syndrome, defined by the presence of pain and thickening in the Achilles tendon.^1^ The lifetime prevalence of AT is 52% in former runners, and the annual incidence is 7%– 9% in current runners.^2^ Onset is typically chronic in nature, although occasionally a patient may present with an acute tendinopathy. There has been a largely successful (although controversial) movement to abandon use of the term “tendinitis” for patients with symptoms that last longer than three months, in recognition of the need to address degenerative structural changes within the tendon (i.e. angiofibroblastic tendinosis) rather than inflammation, which is argued to be rarely or only minimally present in most cases.^3^

The high prevalence among runners points to mechanical loading as an important etiologic factor.^4^ In vitro and in vivo research from the laboratory of Dr Scott and others suggests that periods of mechanical overuse result in subclinical evidence of inflammatory or pseudoinflammatory change.^5-16^ The term “pseudo” is used here to recognize that although cellular inflammation may not be present in an overloaded tendon, we have discovered that tenocytes themselves produce certain inflammatory mediators in response to increased mechanical loading, such as substance P, prostaglandin E2, VEGF, glutamate, and others. Over time, repeated episodes of inflammatory activity have been shown to lead to cumulative changes in the tendon, eventually resulting in the development of tendinosis, paratendonitis or both.^5 17^

A minority of patients with AT have sedentary lifestyles and no identifiable history of mechanical overload, which demonstrates that while external mechanical loading is a risk factor, individual biomechanical^18 19^ and systemic^2^ influences are also likely to be important (as is the case in primary OA). Indeed, multiple risk factors have been identified in small associational studies, including advancing age,^2^ male sex,^2^ high body mass index,^20^ hypercholesterolemia^21^ and the presence of genetic variants of genes related to collagen metabolism.^22 23^

In this introductory section, we review the evidence base for conservative management of chronic midportion AT. Any new treatment must be evaluated in the context of the current evidence base and be tested on a background of the current standard of care.^24^ A plethora of conservative treatments have been suggested to provide relief from, or “cures” for, AT, including nonsteroidal anti-inflammatory drugs (NSAIDs), ultrasound and laser therapies, nutritional supplements, friction massage, and various injection strategies. For the details on the systematic search strategy used for this section, please see the published version of this review in CMAJ, Appendices.^25^

**Is exercise an effective treatment?**

The strongest evidence (multiple, consistent, well controlled trials) supports heavy-load exercise as the cornerstone of managing AT. This form of exercise varies slightly from trial to trial, but the principal goal is to provide a strong, controlled, mechanical force to the Achilles tendon which is tolerable to the patient. This is usually achieved through eccentric (lengthening) muscle activity; for instance, a heel drop over a step. (In contrast, concentric exercise involves a toe raise that would contract the calf muscles.) As the patient’s tolerance of the exercise improves, the load is progressively increased (e.g., by progressing from bilateral to unilateral heel drops, by progressing the degree of dorsiflexion, by adding weights in a backpack or through use of gym equipment). Rompe and colleagues found this type of progressive, slow, heavy-load exercise to be superior to a wait-and-see strategy, with a 60% success rate in the exercise group (95% confidence interval [CI] 41%–79%) compared with 24% in controls who did not receive treatment (95% CI 7%–41%).^26^ Eccentric exercise was found to be superior to a standard exercise program in a multicentre randomized trial by Mafi and colleagues; 82% of the patients who trained with eccentric exercise were satisfied and had returned to their previous activity level after 12 weeks (95% CI 66%–98%), compared with 36% of patients who trained with standard (concentric) exercise (95% CI 16%–56%).^27^ Thus, eccentric exercise induces clinically meaningful effects but may take several weeks or months before a benefit is felt, and not every patient is guaranteed to respond. The potential harms of eccentric exercise include delayed-onset muscle soreness or exacerbation of the tendinopathy (e.g. pain, soreness or swelling). These typically occur when the exercises are performed too quickly, with incorrect biomechanics, or with an increase in load that is too abrupt. There are no published reports of tendon rupture in response to eccentric loading.

**Do orthotics, braces or splints have a positive effect?**

Orthotics may be helpful in conjunction with other modalities of treatment if there is an identifiable malalignment, whereas braces or splints do not appear to improve outcomes in Achilles tendinopathy. One randomized controlled trial of male runners found a significant improvement in pain in patients wearing custom-made orthotics. Of the patients, 89% described a reduction in pain scores of greater than 50% after four weeks of wearing custom insoles (95% CI 69%–100%), compared with 0% in control patients who did not receive treatment (*p* < 0.05).^28^ This trial may have been improperly blinded, because sham insoles were not provided to the control group. Referral to a qualified orthotist should be considered if the biomechanical examination identifies a potentially correctable malalignment. However, there is little evidence to suggest that orthotics alone will be effective in most cases. An inflatable brace was examined in two separate studies. The brace is commercially available (AirHeel [Aircast]) and designed to be worn during activity. An earlier study by Petersen and coworkers could not detect differences between groups and recommended further study.^29^ In a subsequent larger study, Knobloch and colleagues randomly assigned 116 patients to undergo treatment consisting of eccentric training or eccentric training plus prescription of an AirHeel brace. Both groups achieved similar improvements in pain after 12 weeks, which showed that the addition of the AirHeel brace did not improve the success of rehabilitation.^30^ Other authors have advocated use of a night splint, as commonly prescribed for patients with plantar fasciitis. de Vos and colleagues randomly assigned patients to complete a heavy load eccentric training program with or without use of a night splint. After 12 weeks, 63% of patients in the group that received only exercise were satisfied (95% CI 47%–79%), compared with 48% in the night splint group (95% CI 32%–64%), and there were no significant differences in pain or other outcome measures.^31^

**What is the role of physiotherapy**?

Many clinicians recognize that an individualized physiotherapy program that addresses the patient’s specific presentation, loading history and biomechanics, leads to the most effective outcomes when combined with exercise. However, published evidence for these complex interventions is lacking, particularly for longer-term outcomes. One trial examined the short-term effect of physiotherapy, including ice, transverse friction massage, therapeutic ultrasound and exercise (i.e., concentric, eccentric, balance and proprioceptive drills), compared with control (no treatment).^28^ The physiotherapy group (*n* = 11) experienced significant improvements in activity-related pain compared with controls (*n* = 8) (*p* < 0.05). Effect size could not be calculated from the reported data, which made a comparison with other trials that used exercise alone impossible. The trial included only men and used a multimodal intervention that closely approximates interventions currently provided by many sports medicine physiotherapy clinics. Although this improves generalizability, it makes it impossible to distinguish the effective components of the program.

**Nonsteroidal anti-inflammatory drugs**

This review did not identify a randomized controlled trial of NSAIDs that met the inclusion criteria of symptoms lasting longer than three months. One trial of NSAIDs included some patients with chronic Achilles tendinopathy of less than six months’ duration; that trial was rejected for inclusion during the initial literature search (Appendix 1, available at www.cmaj.ca) because it also included patients with acute Achilles tendinopathy. This trial found identical treatment outcomes in 70 patients randomly assigned to receive piroxicam or placebo tablets.^32^ The study is consistent with a lack of effect of NSAIDs in other types of tendinopathy, e.g. tennis elbow.^33^

**Injection therapies**

Several injection therapies for the treatment of chronic Achilles tendinopathy have been examined in small clinical studies. These treatments include corticosteroids, sclerosing therapy, aprotinin, polidocanol, glycosaminoglycan polysulfate, prolotherapy, and platelet-rich plasma.^34-40^ Several studies have reported trends that are consistent with a clinically significant improvement, but are hampered by the small number of patients studied. Fredberg and colleagues randomly assigned 24 patients with midportion Achilles tendinopathy to receive ultrasonography-guided corticosteroid (20 mg triamcinolone) or placebo injections. The authors commented that a significant number of patients treated with corticosteroid injection experienced initial improvements followed by a relapse of symptoms, but it is difficult to extract this information from the trial.^37^ An older study included instances of both acute and chronic Achilles tendinopathy: these authors reported a lack of success with corticosteroid injection for the subset of patients with chronic Achilles tendinopathy.^36^ One randomized controlled trial examined the effect of injection of platelet-rich plasma on patients with Achilles tendinopathy; however, the study included patients with relatively acute conditions (two months’ duration of symptoms) and thus did not meet our a priori inclusion criteria.^41^ Platelet-rich plasma is sometimes considered for instances of chronic, refractory tendinopathy, but there is as yet no evidence from randomized controlled trials to support or refute its use for chronic midportion Achilles tendinopathy^40^. A recent RCT of prolotherapy + eccentric loading exercise (PLE) vs eccentric loading exercise (ELE) reported accelerated recovery in the PLE group; the outcomes were encouraging (7/14 in PLE group achieved an improvement of 20/100 in Victorian Institute of Sports Assessment–Achilles [VISA-A-A] score compared to only 3/15 in the ELE group at 6 weeks)^42^ however this treatment has not yet become part of the first line standard of care.

**Extracorporeal shock wave therapy**

As for injection therapies, the current evidence for shock wave therapy as a treatment for Achilles tendinopathy is promising but inconclusive. Shock wave therapy involves the application of acoustic waves that vary in nature according to the type of device used and the amount of energy and energy flux delivered to the tissue. The evidence that met our criteria rested on two trials from the same group.^26 43^ One trial examined the effect of adding low energy (0.1 mJ/mm^2^) extracorporeal shock wave to a heavy-load eccentric exercise program. Patients who were randomly assigned to receive active shock waves plus exercise fared slightly better in terms of pain (1.5/10 improvement on a visual analogue scale; *p* = 0.0045), activity level (13.5/100 improvement on the VISA-A-A score; *p* = 0.016) and satisfaction with treatment at four months.^18^ Although statistically significant, these average values are rather small; the minimum clinically significant difference for the VISA-A-A score is 15/100, and for pain it is 2/10. However, only mean values were presented – the actual percentage of patients who achieved a clinically significant improvement in each group was not reported.

**Glyceryl trinitrate**

Two randomized controlled trials examined the effect of placing patches of glyceryl trinitrate directly over the painful Achilles midportion. Nitric oxide is essential to achieve normal healing of the tendon after acute injury; therefore, it may also be helpful in promoting repair of chronic injuries.^44^ Paoloni and colleagues randomly assigned 65 patients to receive rehabilitation (focusing on active tendon-loading exercises) plus glyceryl trinitrate for six months, or identical rehabilitation with placebo patches.^45^ The primary outcome measure was patient-rated tendon pain with activity. Four patients did not conclude the trial because of the development of headaches or skin rashes. In those who remained in the trial, there was a statistically and clinically significant decrease in pain with activity (*p* = 0.03). A second study by Kane and coauthors examined the effects of patches of glyceryl trinitrate and eccentric exercise versus placebo patches and eccentric exercise in 40 patients (20 per group).^46^ The main outcome measures were the Ankle Osteoarthritis Scale pain and disability scores. The scores of both groups were significantly decreased at six months, but the average scores were identical in the two groups. Thus, the initial promising results from Paoloni and colleagues’ trial have not been supported by similar studies in other centres.

**What is the prognosis?**

Although tendinopathy may seem a relatively benign soft tissue pathology, recovery often follows a prolonged time course, even with ideal treatment. Because traditional surgical management has only a 50% rate of success,^47^ it is essential to ensure that the best conservative management has been offered before consideration of surgery. The recalcitrant nature of chronic Achilles tendinopathy was documented by Rompe and colleagues, who noted that there was a slow but gradual improvement in the average load-induced pain score from 7.9/10 to 5.9/10 over the course of four months in patients not receiving treatment. After this period, only 24% of these patients reported themselves as much improved or cured.^26^ In the long term, the prognosis of chronic Achilles tendinopathy is more favourable. At eight-year follow-up in one study of 83 patients, 80% of patients were back to full physical activities, whereas 20% reported that their physical activity was still impaired as a result of the Achilles tendinopathy.^48^

# Intramuscular stimulation – rationale for its use in chronic Achilles tendinopathy

Currently, there are no clinical or laboratory studies on the effect of IMS on Achilles tendinopathy. However, Dr Gunn has convincingly presented the rationale for IMS therapy for this condition.^49^ IMS practitioners have noted that chronic pain conditions like tendinopathy are almost always accompanied by areas of chronic muscle tension and shortening that may create increased strain in associated tendons.^49^ Recent work has established that strain, rather than load per se, is the primary driver of tendon microdamage during cyclic loading.^50^ Additionally, focal areas of muscle thickening could create nonhomogenous strains and/or shearing within the Achilles tendon.^51^ During a typical running gait cycle (males running at 12 km/hr), the Achilles tendon is subjected to strains in the range of 3.8 ± 2%^52^, meaning that the tendon is functioning in the linear area of the load-displacement curve, just under the yield point where more substantial fatigue damage begins to accumulate (~5%)^50 53^. Muscle shortening as a whole, or localized areas of shortening within the whole muscle, would lead to slight but potentially significant increases in the strain experienced by and within the tendon, whereby the tendon could experience injuriously higher peak strains (i.e. above 5%). If IMS can alleviate localized foci of muscle shortening as noted clinically, then the strain levels within the tendon may be lessened. This would tend to minimize ongoing fatigue damage experienced by the tendon (for instance, when a patient engages in running or jumping activities).

Sustained whole muscle stretching is frequently used by therapists when treating tendinopathies^.(e.g 54)^ However, localized areas of shortened muscle (i.e. the tender/painful bands or “trigger points” targeted by IMS) clinically do not respond well to this type of stretching. Muscle shortening in the gastrocnemius and soleus muscles is clinically observed as a reduction in dorsiflexion range of motion (ROM) with the knee straight or bent. Achilles tendinopathy has been associated (in cross-sectional case control studies) with a reduced range of dorsiflexion ROM.^19^ Indeed, dorsiflexion ROM has been used as a secondary outcome measure in clinical trials of Achilles tendinopathy.^55^ IMS may effectively and efficiently restore normal DF ROM by releasing tight muscle bands.

IMS may also have a role to play in restoring a neurotrophic effect to tendon tissue to normalise collagen turnover and tendon strength.^49^ Additionally, direct needling of tendon (a technique included in the IMS armament of needling procedures) can stimulate a local inflammatory response and the release of growth factors such as platelet-derived growth factor.^56^ IMS may therefore have a role to play in stimulating healing in tendinosis.

# Study Design

This is a prospective, single blind, randomized controlled trial with three groups. All groups will receive standard 12 week physiotherapy program provided by LS, including a standardized and progressive eccentric training and strengthening program. The first group will receive IMS (detailed below), while the second will receive an equivalent number of sham (superficial non-intramuscular) needling sessions. The third receives the progressive exercise training program alone. Patients will be recruited from the clinical practice of LS and colleagues; additionally, the study will be advertised through LS’s and AS’s contacts in the IMS, UBC, Physiotherapy Association of BC, and Vancouver Coastal Health networks. After providing informed consent and being screened for inclusion/exclusion criteria, diagnosis will be confirmed by physical examination. Inclusion criteria include subjects 19 to 60 years of age, fluent in English, with a 3 month minimum symptom duration, evidence of midportion Achilles tendinopathy on physical examination and tendinosis on ultrasound; and objective signs by Gunn IMS assessment of neuropathic change in the L5-S2 segmental levels including the presence of taut muscle bands amenable to IMS.^49^ Tight bands palpated in muscles paraspinally (dorsal rami) from T1 down and peripherally (ventral rami) from T12 down will also be treated in the IMS group, but participants without findings in the L5-S2 levels will be excluded to limit variability among subjects. Exclusion criteria include IMS contraindications (infection in the area, pregnancy, bleeding disorders, history of bacterial endocarditis, post-surgical implant in last four to six months and major surgery in the last three months), previous treatment with IMS or TCMA (for blinding purposes); true leg length difference of greater than ½” previous corticosteroid injections or recent fluoroquinolone use and systemic inflammatory disease. Those demonstrating the presence of other syndromes that cause pain in and around the Achilles tendon will be excluded by history-taking and physical examination: partial tearing, posterior ankle impingement/ os trigonum syndrome, tenosynovitis, dislocation of the peroneal or other plantar flexor tendons, irritation or neuroma of the sural nerve or insertional Achilles pain. Patients will be randomized by the research assistant (Heather Denton) who is blinded to the patient’s baseline data using a random number generator (SPSS v.16.0).

## Study Team and Roles

**Heather Denton**

Conduct initial phone screen (with Lyndal Solomons and Alex Scott).

After final eligibility screen by Lyndal Solomons and informed consent obtained, consult random number generation table and record subject group allocation, ensure confidentiality of files (must be in a locked cabinet accessible only by the study team).

Collate initial subject files and deliver to locked cabinet at Kinetic clinic.

Prepare patient exercise diary with appropriate dates filled in.

Collect hard copies of data (VISA-A forms and Global Improvement forms completed by study participants, ankle ROM forms completed by MB, treatment attendance data logged by LS) from locked cabinet at Kinetic and enter into Master Tracking Sheet on password-protected computer.

Monitor patient flow through study, ensure all appointments are booked and confirmed when they happen, including assessment by MB and UTC scans by LS, as per Master Tracking Sheet.

Maintain unblinding log.

Contact participants re 1 year follow-ups.

Send de-identified data (e.g. copies of Master Tracking Sheet) to Alex Scott or designate upon request.

**Lyndal Solomons**

Oversee recruitment strategy, distribution of posters and study materials. Conduct initial phone screen (with Alex Scott and Heather Denton).

Conduct physical assessment screen.

Obtain informed consent. (Documents to be stored in locked cabinet at Kinetic for collection by HD).

Create tracking sheet for ankle range measures, VISA-A questionnaires (n= 4 x 42 = 168), Global Improvement scales (n= 4 x 42 = 168), and unblinding sheets for Margaret Bruce and deliver to locked cabinet at Kinetic.

Collect ultrasound tissue characterization (UTC) images.

Interpret UTC images (with Alex Scott).

Provide dry needling and exercise treatments and progressions.

Report unexpected events (per UBC Nov 11 2012 policy) to Alex Scott.

Track study expenditures.

Remain blinded to VISA-A / Global Improvement scores / ROM scores.

Record patient attendance dates and store in locked cabinet for Heather Denton to access and record on Master Tracking Sheet.

Ensure data stored securely.

Deliver data from Canopy to Kinetic for collection by HD.

Primary contact in case of adverse event.

**Margaret Bruce**

Conduct blinded ankle ROM assessments and deliver questionnaires to participants at entry into study, after 6 weeks of treatment, after 12 weeks of treatment and 1 year after entry into study.

Direct participants in completing questionnaires ie participant to enter study ID # and time point in study (entry into study, after 6 weeks of treatment, after 12 weeks of treatment and at 1 year after entry into study)and deliver to front desk for storage in locked cabinet.

Remain blinded to participant allocation. Report patient unblinding.

**Alex Scott**

Conduct initial phone screen (with Lyndal Solomons and Heather Denton).

Interpret UTC images (with Lyndal Solomons).

Maintain study ethics.

Review unanticipated problems, report to CREB if necessary as soon as reasonably possible.

Data audits.

Financial reporting to UBC.

Liase with UBC statistical services.

Retain hard copies of patient data in secure area for 7 years following study publication.

**UBC statistical services**

Analyze the data at midpoint of recruitment

Statistical analysis after target number (n=42) have passed 3 month time point.

**Study Flow Chart**

**Physical Examination**

Physical examination will incorporate a standard physiotherapy assessment, a standard IMS assessment, and a specific assessment of the Achilles tendon itself by an experienced practitioner (LS). The physiotherapy assessment will include examination of the lumbar spine, pelvis, hip, knee, ankle and foot as relevant. Examination of these regions, as appropriate, will include: range of motion assessment; muscle length tests; manual muscle testing; observation of alignment/symmetry; and foot architecture (subtalar or forefoot varus; cavus or pronated foot). Biomechanical issues that could be expected to have an impact on the magnitude of strain delivered to the Achilles tendon will be addressed on an individual basis.^57 58^

The IMS assessment is targeted to observe for the presence of neuropathic change, as operationally defined by Gunn, and will be derived from the general and region-specific examination protocol he describes.^49^ Manifestations of neuropathic change that may be observed clinically include: sensory manifestations (hyperpathia and allodynia), autonomic manifestations (vasoconstriction, sudomotor activity, pilomotor reflex, trophedema and trophic changes in skin, hair and nails) and motor manifestations (muscle shortening, limitation of joint range and enthesopathy). When neuropathic change is present, there is typically a mixture of sensorimotor and autonomic disturbances evident, with findings grouped in a segmental distribution that indicate the affected spinal level(s) and muscles involved. Aspects of the Gunn IMS examination that may not already have been assessed in the standard physiotherapy assessment include: observation of posture and gait, any obvious muscle wasting or dermatomal hair loss; quick screening tests; neurological examination of the L2 to S1 segments in terms of power and reflexes; straight leg raise test of neural provocation; and specific motor, sensory and autonomic neuropathic assessments. Observation of posture allows for the detection of variations in spinal curvature (scoliosis or anomaly of the normal kyphotic and lordotic curvatures) and asymmetries in the shoulder or pelvic girdles. Quick tests include Trendelenburg’s sign (to assess for weakness in the gluteal muscles, especially gluteus medius), squat (to screen the hip and knee joints), and FABERE/Patrick’s test for limitation of hip joint range. The regional part of the IMS examination for pain in the calf, in this investigation, will be looking specifically for patterns of neuropathic change in the L5-S2 segments. These levels are of key significance as they comprise the nerve supply to the gastrocnemius and soleus muscles i.e. S1 and S2 from the tibial nerve (L4-S3)^59^, and to the Achilles tendon itself i.e. the sural nerve (derived mostly from the S1 segment but also from L5 and S2).^60^ Although spinally the focus will be on the lumbar region, assessment (and treatment) will extend into the thoracic spine as recommended by Dr Gunn.^49^ The spine will be palpated for prominent and tender spinous processes which may be an indicator of dysfunction at that level. Hyperpathia will be tested paraspinally by running a pin down the body, crossing dermatomes. Skin temperature will be palpated to assess for any obvious coolness (vasoconstriction). The Skin Rolling and Matchstick tests for trophedema will be conducted. Observation will be made for the presence of: a peau d’orange effect (trophedema); excessive sweating (sudomotor activity); “goosebumps” (pilomotor reflex); and trophic changes in skin, hair (dermatomal loss) and nails. The lumbar back muscles, innervated by the dorsal rami of the spinal nerves, including the long polysegmental muscles (the thoracic components of the longissimus and iliocostalis lumborum) and the polysegmental muscles that attach to the lumbar vertebrae (the multifidus and the lumbar components of the longissimus and iliocostalis)^61^ will be assessed for the presence of painful tight bands. The deep muscles may only be fully assessed for signs of hypersensitivity by penetration with a needle. The peripheral muscles, innervated by the ventral rami of the spinal nerves, will also be assessed for the presence of painful tight bands.

Subjects with Achilles tendinopathy who do not demonstrate region-specific signs as defined by this assessment will be excluded from the study to reduce potential variability in treatment response.

The Achilles tendon will be assessed for the presence of tenderness on palpation of the mid-portion, with or without thickening of this region, and pain on tendon loading.^25 62^ Tendon loading will be achieved by asking subjects to perform heel raises (double or single), hopping or hopping forward as required to assess for a pain response.^62^

**IMS treatment group**

Intramuscular Stimulation (IMS) involves the insertion of fine sterile needles into tender and painful points in tight muscle bands (hypothesized in the IMS literature to be caused by reduced nerve supply).^49^ Needles inserted into muscles create an electrical discharge.^49^ In patients with chronic musculoskeletal pain syndromes, this discharge is strongly exaggerated and results in twitches, fasciculations or sustained contractions in the targeted muscle.^49^ Patients will feel these various muscular reactions, with the sustained contractions producing a distinctive deep aching sensation that is cramp-like in quality.^49^ This sensation is analogous to the Teh Ch’i or Deqi Phenomenon in Traditional Chinese Medicine Acupuncture (TCMA) literature.^49^ Treatment will be guided by the findings of the assessment, which will have paid particular attention to signs in groupings of muscles supplied by both the dorsal and ventral rami of the same segmental spinal level. Muscles in the periphery that have demonstrated tight bands or reduced muscle lengths will be treated with IMS by one of the investigators (LS). Muscles at levels of the spine that have demonstrated tight muscle bands, signs of trophedema or tender, prominent spinous processes will also be treated. Those muscles that respond to needle penetration with sustained contractions or “needle grasp”, such that resistance is felt on attempting to withdraw the needle, will be left in situ until the muscle releases. This may occur quickly, but may be expected to take up to twenty minutes.^49^ Those muscles that respond with only fasciculations or twitches may be treated with extra stimulation by “fanning” or “twirling”, depending on patient tolerance, but needles will then be promptly removed. Needles will also be briefly inserted into the Achilles tendon to produce a local inflammation-healing response.^49^ The IMS group will receive treatment once a week for the first six weeks of the trial and once every two weeks for the remainder, resulting in nine treatments overall.

**Sham needling**

The appropriate control for acupuncture or needling studies is always controversial, as the act of needling nonspecifically, even superficially, can induce a variety of physiological responses as well as a variable level of placebo response.^63^ Nonetheless, recently the importance of including both sham needling and no-treatment controls has been emphasized (c.f. section 6, and figure 1, in ^63^), and considered superior to non-penetrating “placebo needles.^63^ In the sham needling group, eight acupuncture needles will be inserted into the dermis (1-2 mm) of the buttock, posterior thigh and calf and will be left *in situ* for ten minutes. Points will be chosen that do not mimic either IMS or TCMA techniques. To avoid mimicking IMS treatment the needles will be inserted away from any tight muscle bands that may be present and will be inserted superficially so that no muscle penetration occurs. To avoid mimicking TCMA treatment, meridians in the treatment area and points used to treat heel pain specifically will be avoided, the depth of needle insertion will be superficial, and no “deqi” sensation will be elicited. Meridians in the area of the buttock (the bladder and Du meridians) are in the midline of the buttock and medial to this and so will not correspond with needle points used in IMS (more lateral). In the posterior leg, the bladder meridian is in the midline to the level of the inferior border of the gastrocnemius muscle and so will not correspond with IMS-targeted muscle banding (medial and lateral of the midline). Below the gastrocnemius muscle the bladder meridian moves laterally and so will not correspond with IMS needling of the Achilles tendon itself. No sham needles will be used in the area of the lower back or sacrum as the IMS points and TCMA points correspond more closely in this region. Particular care will be taken to avoid needling in the area of TCMA points used to treat heel pain specifically in the treatment area, these being: Bladder 57 (inferior edge of gastrocnemius muscle in midline) and Bladder 60 (in the depression midway between the lateral malleolus and the Achilles tendon, level with the high point of the bone), Kidney 4 (medial ankle, in the depression anterior to the angle formed by the Achilles tendon and the calcaneum), Gall Bladder 34 - “the physio point” (in the depression anterior and inferior to the head of the fibula) and Bladder 40 (at the mid-point of the transverse crease of the popliteal fossa).^64^

**Training program**

All three groups will undertake an exercise programme designed to initially maintain and then, as tolerated, increase the ability of the muscle-tendon unit and kinetic chain to absorb load. There will be four stages in this programme andEccentric exercises consisting of gastrocnemius-soleus-Achilles tendon complex loading (ELE) will be incorporated at each stage Eccentric loading has been demonstrated to be the most consistently effective conservative intervention in the treatment of mid-portion Achilles tendinosis.^25^ The program will be modeled on those developed by Alfredson and colleagues^27 65^ and Silbernagel and colleagues^48 66^. Eccentric exercises will be prescribed in a progressive manner for the twelve week duration of the trial. Exercise progression will be as follows: two-legged very slow (Stage 1a) to slow (Stage 1b) eccentric exercise to plantargrade (on flat ground) adding load with weighted backpack or resistance equipment as available (Stage 1a progressing to Stage 1b). The next level of exercises (Stage 2) will progress to single-legged slow eccentric exercise into dorsiflexion adding load with weighted backpack or resistance equipment as available Further progression of loading will then be achieved by introducing speed to previously prescribed exercises using only body weight as resistance (Stage 3). Those participants who reach Stage 4 will be prescribed sports specific exercises aimed at increasing the capacity of the muscle-tendon unit to absorb elastic loading to functional levels. Stage 1 exercises will be prescribed two to three times a day. Stage 1b exercises will be prescribed once a day, every other day. Stage 2 exercises will be prescribed every other day. Stage 3 exercises will be prescribed once a day every third day. All subjects on progression form Stage 1 to Stage 2 of the rehabilitation programme will have a scheduled rest day. On commencement of Level 2 exercises, Stage 1 and 2 level exercises will be alternated for 6 days of the week and the seventh day will be allocated a s a rest day. Once Stage 3 exercises are added, Stage 1, 2 and 3 exercises will be alternated for 6 days of the week and the seventh day will be allocated a s a rest day.

All subjects will be entered into the rehabilitation programme at Stage 1. Subjects will be progressed based on a twenty four hour response to exercise, specifically there must not be pain or stiffness the morning after undertaking the exercise. Pain during exercises will be allowed as long as it is not more than 5/10 pain on a zero to ten scale, where zero is no pain at all and ten is the worst possible imaginable pain. Through all four stages, those subjects in the IMS group and the sham needling group will be advised to rest from their ELE and exercise generally (including stretches) after their treatment for the remainder of the day and the entire next day. From Stage 2 on, this rest day will be scheduled to coincide with the day seven rest day that all groups will observe. They will be instructed to recommence their exercise program the morning after. This is standard advice given to patients receiving IMS treatment.

All subjects will be advised that they may participate in pain-provoking activity, such as running, as long as their pain does not reach greater than 2-3/10 (Visual Analogue Scale) during the activity and as long as pain has returned to baseline by the following morning. The provocation of this level of pain up to 5/10 VAS during rehabilitation has been shown to not hinder recovery.^48 66^

The exercise technique and progression will be reviewed every two weeks until 12 weeks (6 reviews total).

**Outcome measures**

The primary outcome measure for which the study is powered is the 12 week VISA-A score - a well validated and reliable disease-specific outcome measure, which also includes an activity-related pain scale.^67^ Secondary outcome measures include treatment success/failure, and muscle length (dorsiflexion ROM). Patients will rate themselves from 0 (very much worse) to 6 (very much improved). Scores of very much improved or much improved will be categorized as successes.^68^ Dorsiflexion range of motion with knee bent and straight will also be measured using an inclinometer, giving an indication of the effect of IMS and exercise vs exercise alone on muscle extensibility. These measures will be taken at 0 and 12 weeks, as well as at 52 weeks.[[1](#_ENREF_1)] After 12 weeks and 6 months of intervention, UTC scanning will be conducted to determine, as a pilot study, whether there is any subjective indication of a potential effect of IMS on tissue remodeling that could be pursued with a mechanistic study in future.

Muscle length measures will be made according to the protocol described by Norkin and White^69^ and Kendall and colleagues^70^ by a physiotherapist (MB) unaware of the treatment allocation. The two-joint plantarflexors (gastrocnemius and plantaris) will be measured in supine with the knee extended. The one-joint plantarflexors will also be measured in supine but with the hip and knee flexed 90 degrees or more to make the two-joint plantar flexors slack across the knee joint. The ankle will be passively dorsiflexed and the angle achieved measured by goniometry. The fulcrum will be centred over the lateral aspect of the lateral malleolus, the proximal arm will be aligned with the lateral midline of the fibula, using the head of the fibula for reference, and the distal arm will be positioned parallel to the lateral aspect of the fifth metatarsal. The ankle will be dorsiflexed to end-of-range (firm muscular end-feel) by pushing upward across the plantar surface of the metatarsal heads, without allowing the foot to rotate into inversion or eversion. Intratester reliability in measuring passive ankle dorsiflexion by goniometry has been shown to have an Intraclass Correlation Coefficient (ICC) of 0.91 for more experienced therapists.^71^ MB has had over three decades of musculoskeletal clinical experience.

# Statistical analysis

Based on the published literature, we estimate that the no-needling and sham needling groups will improve on average by 20 VISA-A points after 12 weeks (due to the effects of exercise-based physiotherapy management),^25^ and we hypothesize that the IMS group will improve by a further 15 points (the minimum clinically significant difference).^42^ The SD of a single VISA-A score is 15^67^, and correlation between repeated measurements on the same patient after 1 week is 0.80.^67^ This makes the SD for the difference between 2 measurements on the same patient approximately 9.5. To detect these effects in a one-way ANOVA model with a residual standard deviation of 9.5 with power 95%, a sample size of 11 patients is needed in each group for a total of 33 patients. Assuming a 20% drop-out rate,^43^ 42 patients will initially be recruited and enrolled. The primary analysis will be a one-way Analysis of Variance with the response being the change in patients VISA-A score at 12 weeks. Secondary analyses will be performed on the change in patients VISA-A score at 6 months and 12 months from baseline, and on changes in dorsiflexion range of motion and global improvement scale. Inter-group post-hoc tests will be conducted in consultation with UBC statistical consulting services (Rick White, manager). Intention to treat analysis will be employed. If a clinically significant effect of IMS in Achilles tendinopathy exists, this pilot study has a 95% chance of successfully detecting it. If there is no clinically significant effect of IMS in Achilles tendinopathy, this study has a 95% chance of reaching this conclusion.

# Scientific rigor

If the results indicate the existence of a clinically significant effect of IMS, then a larger study with greater attention to mechanisms would be indicated in future. By including both control groups (exercise alone, sham needling), the study design will allow appropriate conclusions to be drawn. If an exercise alone group is not included and if the sham needling and IMS groups were equivalent, then we would not know if IMS and sham needling are equally effective or equally ineffective. Conversely, if we did not have a sham needling group and if the IMS group were superior to exercise group, then we could not conclude that IMS is effective – the effects may have been due to a non-specific effect of needling.^63^ After 50% of the patients, have been enrolled, one researcher (AS) will check the patient baseline data to ensure that the randomized groups are equivalent (in terms of symptom duration, gender, age, baseline VISA-A). If necessary, the subsequent randomization will be weighted in consultation with UBC statistical consulting services. The RCT will be reported and conducted according to the guidelines of the CONSORT (CONsolidated Standards Of Reporting Trials) statement 2010, and ensuring that a score of 10/11 is achieved on the PEDro score (physiotherapy evidence database). It is not possible to blind the treating therapist therefore a score of 11/11 is not possible.

# Ability to do the work

Lyndal Solomons is an experienced IMS practitioner. Lyndal has fourteen years of experience working as a physiotherapist and holds a Bachelor of Applied Science in Physiotherapy and a Master of Manual Therapy, has certification in Gunn IMS and is a member of iSTOP. Given the prevalence of Achilles tendinopathy and the offer of free physical therapy treatment, recruitment will not pose any threat to the success of this study. Dr Scott is an Assistant Professor in Physical Therapy with protected time for clinical research (Michael Smith Scholar award) with postgraduate AFCI training (Acupuncture Foundation of Canada Institute). Not only are we ideally placed to conduct this project in clinical and research skills, we are very well networked with professional organizations and as such the results can be immediately disseminated to target groups including iSTOP members (please see letter of support fom iSTOP education director), UBC MPT students, PABC members, the national and international physiotherapy communities (Canadian Physiotherapy Association and International Federation of Orthopaedic and Manipulative Physiotherapists), Worksafe BC healthcare providers, as well as other research interest groups (e.g. Instititute of Musculoskeletal Health and Arthritis). Dr Scott is a member of the PABC-UBC Tendinopathy Task force which develops and distributes clinical resource materials to B.C. clinicians. Please see Dr Scott’s CV for other details of knowledge translation work.

**Limitations**

This is a relatively small clinical trial (target starting population, n=42). However, we note that this population is consistent with other recent clinical trials in high impact Sports Medicine journals for midportion AT (e.g. Yelland et al, 3-arm trial, similar design to ours, starting n=43^42^). Our sample size was arrived at via power calculation conducted by UBC statistical services. Although the study is powered to detect clinically significant differences between treatment groups, its generalizability may be limited by the treatment population (recreational athletes with tendinosis on US and with L5-S2 region-specific signs identified by an IMS assessment). If the trial demonstrates a clinical effect of IMS on chronic midportion AT in these patients, further study with other populations (e.g. elite athletes or workers) would be indicated. Additionally, the inter- and intra-observer reliability of the IMS assessment procedure has not been documented to our knowledge; in this case, a single experienced IMS practitioner will conduct the assessments. A further limitation is that it is not possible to create a truly sham IMS treatment, as the technique creates a strong, distinctive sensation. Our compromise is to use sham superficial (skin) needling to control for at least a portion of the expected placebo response, however there are no published data on the magnitude of placebo response to needling for AT. A further limitation is that the groups which receive needling (sham or IMS) vs exercise alone have slightly different visit numbers, and that the number of exercise sessions will be slightly fewer for the needling groups (they are typically asked to refrain from exercise for 24 to 48 hours after needling sessions). However, the magnitude of these differences is not enough to cause a meaningful clinical difference in our experience.

**Significance**

This study will be the first iSTOP-UBC collaborative study and the first RCT examining the effect of IMS on AT, the vast majority of which are associated with taut muscle bands that are highly amenable to IMS. If successful, this study would be likely to stimulate further research by ourselves or other groups, e.g. examining the effects of IMS on other tendinopathies such as the lateral elbow. Dr Gunn has argued convincingly that the rationale for IMS in chronic tendinopathies involves the ability to relieve associated muscle shortening, (e.g. ^49^ p 265,266) but this hypothesis has not yet been tested by a blinded assessor as we propose to do here. The prevalence of AT is high and its impact on physical activity is significant, with up to 20% of patients experiencing pain and activity limitation after 5 years despite optimal management^48^. This proposal addresses a recognized gap between clinical experience and research evidence, and the results will thus be directly relevant and of potentially high impact to practising clinicians and their patients.

**REFERENCES**

1. Ryan M, Wong A, Taunton J. Favorable outcomes after sonographically guided intratendinous injection of hyperosmolar dextrose for chronic insertional and midportion achilles tendinosis. *AJR Am J Roentgenol* 2010;194(4):1047-53.

2. Maffulli N, Wong J, Almekinders LC. Types and epidemiology of tendinopathy. *Clin Sports Med* 2003;22(4):675-92.

3. Fredberg U. Tendinopathy--tendinitis or tendinosis? The question is still open. *Scand J Med Sci Sports* 2004;14(4):270-2.

4. Maffulli N, Waterston SW, Squair J, et al. Changing incidence of Achilles tendon rupture in Scotland: a 15-year study. *Clin J Sport Med* 1999;9(3):157-60.

5. Andersson G, Backman LJ, Scott A, et al. Substance P accelerates hypercellularity and angiogenesis in tendon tissue and enhances paratendinitis in response to Achilles tendon overuse in a tendinopathy model. *Br J Sports Med* 2011.

6. Andersson G, Forsgren S, Scott A, et al. Tenocyte hypercellularity and vascular proliferation in a rabbit model of tendinopathy: contralateral effects suggest the involvement of central neuronal mechanisms. *Br J Sports Med* 2010.

7. Attia M, Scott A, Duchesnay A, et al. Alterations of overused supraspinatus tendon: a possible role of glycosaminoglycans and HARP/pleiotrophin in early tendon pathology. *J Orthop Res* 2012;30(1):61-71.

8. Backman LJ, Fong G, Andersson G, et al. Substance P is a mechanoresponsive, autocrine regulator of human tenocyte proliferation. *PLoS One* 2011;6(11):e27209.

9. Scott A, Alfredson H, Forsgren S. VGluT2 expression in painful Achilles and patellar tendinosis: evidence of local glutamate release by tenocytes. *J Orthop Res* 2008;26(5):685-92.

10. Scott A, Bahr R. Neuropeptides in tendinopathy. *Front Biosci* 2009;14:2203-11.

11. Scott A, Cook JL, Hart DA, et al. Tenocyte responses to mechanical loading in vivo: a role for local insulin-like growth factor 1 signaling in early tendinosis in rats. *Arthritis Rheum* 2007;56(3):871-81.

12. Scott A, Danielson P. An emerging role for angiogenesis in tendinopathy. *European Musculoskeletal Review* 2009;4(1):75-76.

13. Scott A, Khan KM, Heer J, et al. High strain mechanical loading rapidly induces tendon apoptosis: an ex vivo rat tibialis anterior model. *Br J Sports Med* 2005;39(5):e25.

14. Scott A, Lian O, Bahr R, et al. VEGF expression in patellar tendinopathy: a preliminary study. *Clin Orthop Relat Res* 2008;466(7):1598-604.

15. Scott A, Lian O, Bahr R, et al. Increased mast cell numbers in human patellar tendinosis: correlation with symptom duration and vascular hyperplasia. *Br J Sports Med* 2008;42(9):753-7.

16. Scott A, Lian O, Roberts CR, et al. Increased versican content is associated with tendinosis pathology in the patellar tendon of athletes with jumper's knee. *Scand J Med Sci Sports* 2008;18(4):427-35.

17. Sullo A, Maffulli N, Capasso G, et al. The effects of prolonged peritendinous administration of PGE1 to the rat Achilles tendon: a possible animal model of chronic Achilles tendinopathy. *J Orthop Sci* 2001;6(4):349-57.

18. Ryan M, Grau S, Krauss I, et al. Kinematic analysis of runners with achilles mid-portion tendinopathy. *Foot Ankle Int* 2009;30(12):1190-5.

19. Clement DB, Taunton JE, Smart GW. Achilles tendinitis and peritendinitis: etiology and treatment. *Am J Sports Med* 1984;12(3):179-84.

20. Gaida JE, Cook JL, Bass SL. Adiposity and tendinopathy. *Disabil Rehabil* 2008;30(20-22):1555-62.

21. Gaida JE, Alfredson L, Kiss ZS, et al. Dyslipidemia in Achilles tendinopathy is characteristic of insulin resistance. *Med Sci Sports Exerc* 2009;41(6):1194-7.

22. Mokone GG, Schwellnus MP, Noakes TD, et al. The COL5A1 gene and Achilles tendon pathology. *Scand J Med Sci Sports* 2006;16(1):19-26.

23. Posthumus M, Collins M, Cook JL, et al. Components of the transforming growth factor-β family and the pathogenesis of human Achilles tendon pathology: a genetic association study *Rheumatology* 2010;Online publication April 1.

24. Audige L, Ayeni OR, Bhandari M, et al. A practical guide to research: design, execution, and publication. *Arthroscopy* 2011;27(4 Suppl):S1-112.

25. Scott A, Huisman E, Khan K. Conservative treatment of chronic Achilles tendinopathy. *CMAJ* 2011;183(10):1159-65.

26. Rompe JD, Nafe B, Furia JP, et al. Eccentric loading, shock-wave treatment, or a wait-and-see policy for tendinopathy of the main body of tendo Achillis: a randomized controlled trial. *Am J Sports Med* 2007;35(3):374-83.

27. Mafi N, Lorentzon R, Alfredson H. Superior short-term results with eccentric calf muscle training compared to concentric training in a randomized prospective multicenter study on patients with chronic Achilles tendinosis. *Knee Surg Sports Traumatol Arthrosc* 2001;9(1):42-7.

28. Mayer F, Hirschmuller A, Muller S, et al. Effects of short-term treatment strategies over 4 weeks in Achilles tendinopathy. *Br J Sports Med* 2007;41(7):e6.

29. Petersen W, Welp R, Rosenbaum D. Chronic Achilles tendinopathy: a prospective randomized study comparing the therapeutic effect of eccentric training, the AirHeel brace, and a combination of both. *Am J Sports Med* 2007;35(10):1659-67.

30. Knobloch K, Schreibmueller L, Longo UG, et al. Eccentric exercises for the management of tendinopathy of the main body of the Achilles tendon with or without the AirHeel Brace. A randomized controlled trial. A: effects on pain and microcirculation. *Disabil Rehabil* 2008;30(20-22):1685-91.

31. de Vos RJ, Weir A, Visser RJ, et al. The additional value of a night splint to eccentric exercises in chronic midportion Achilles tendinopathy: a randomised controlled trial. *Br J Sports Med* 2007;41(7):e5.

32. Astrom M, Westlin N. No effect of piroxicam on achilles tendinopathy. A randomized study of 70 patients. *Acta Orthop Scand* 1992;63(6):631-4.

33. Bisset L, Paungmali A, Vicenzino B, et al. A systematic review and meta-analysis of clinical trials on physical interventions for lateral epicondylalgia. *British journal of sports medicine* 2005;39(7):411-22; discussion 11-22.

34. Alfredson H, Ohberg L. Sclerosing injections to areas of neo-vascularisation reduce pain in chronic Achilles tendinopathy: a double-blind randomised controlled trial. *Knee Surg Sports Traumatol Arthrosc* 2005;13(4):338-44.

35. Brown R, Orchard J, Kinchington M, et al. Aprotinin in the management of Achilles tendinopathy: a randomised controlled trial. *Br J Sports Med* 2006;40(3):275-9.

36. DaCruz DJ, Geeson M, Allen MJ, et al. Achilles paratendonitis: an evaluation of steroid injection. *Br J Sports Med* 1988;22(2):64-5.

37. Fredberg U, Bolvig L, Pfeiffer-Jensen M, et al. Ultrasonography as a tool for diagnosis, guidance of local steroid injection and, together with pressure algometry, monitoring of the treatment of athletes with chronic jumper's knee and Achilles tendinitis: a randomized, double-blind, placebo-controlled study. *Scand J Rheumatol* 2004;33(2):94-101.

38. Shrier I, Matheson GO, Kohl HW, 3rd. Achilles tendonitis: are corticosteroid injections useful or harmful? *Clin J Sport Med* 1996;6(4):245-50.

39. Sundqvist H, Forsskahl B, Kvist M. A promising novel therapy for Achilles peritendinitis: double-blind comparison of glycosaminoglycan polysulfate and high-dose indomethacin. *Int J Sports Med* 1987;8(4):298-303.

40. Engebretsen L. IOC Consensus Statement on the use of platelet-rich plasma (PRP) in sports medicine. 2010.

41. de Vos R, Weir A, van Schie H, et al. Platelet-Rich Plasma Injection for Chronic Achilles Tendinopathy. *JAMA* 2010;303(2):144-49.

42. Yelland MJ, Sweeting KR, Lyftogt JA, et al. Prolotherapy injections and eccentric loading exercises for painful Achilles tendinosis: a randomised trial. *Br J Sports Med* 2011;45(5):421-8.

43. Rompe JD, Furia J, Maffulli N. Eccentric loading versus eccentric loading plus shock-wave treatment for midportion achilles tendinopathy: a randomized controlled trial. *Am J Sports Med* 2009;37(3):463-70.

44. Murrell GA, Szabo C, Hannafin JA, et al. Modulation of tendon healing by nitric oxide. *Inflamm Res* 1997;46(1):19-27.

45. Paoloni JA, Appleyard RC, Nelson J, et al. Topical glyceryl trinitrate treatment of chronic noninsertional achilles tendinopathy. A randomized, double-blind, placebo-controlled trial. *J Bone Joint Surg Am* 2004;86-A(5):916-22.

46. Kane TP, Ismail M, Calder JD. Topical glyceryl trinitrate and noninsertional Achilles tendinopathy: a clinical and cellular investigation. *Am J Sports Med* 2008;36(6):1160-3.

47. Tallon C, Coleman BD, Khan KM, et al. Outcome of surgery for chronic Achilles tendinopathy. A critical review. *Am J Sports Med* 2001;29(3):315-20.

48. Silbernagel KG, Brorsson A, Lundberg M. The majority of patients with Achilles tendinopathy recover fully when treated with exercise alone: a 5-year follow-up. *Am J Sports Med* 2011;39(3):607-13.

49. Gunn CC. *The Gunn Approach to the Treatment of Chronic Pain: Intramuscular Stimulation for Myofascial Pain of Radiculopathic Origin, 2^nd^ edn*. Edinburgh, London, New York, etc.: Churchill Livingstone, 1989.

50. Schechtman H, Bader DL. Fatigue damage of human tendons. *J Biomech* 2002;35(3):347-53.

51. Arndt AN, Komi PV, Bruggemann GP, et al. Individual muscle contributions to the in vivo achilles tendon force. *Clin Biomech (Bristol, Avon)* 1998;13(7):532-41.

52. Farris DJ, Trewartha G, McGuigan MP. The effects of a 30-min run on the mechanics of the human Achilles tendon. *Eur J Appl Physiol* 2012;112(2):653-60.

53. Schechtman H, Bader DL. In vitro fatigue of human tendons. *J Biomech* 1997;30(8):829-35.

54. Martinez-Silvestrini JA, Newcomer KL, Gay RE, et al. Chronic lateral epicondylitis: comparative effectiveness of a home exercise program including stretching alone versus stretching supplemented with eccentric or concentric strengthening. *J Hand Ther* 2005;18(4):411-9, quiz 20.

55. Neeter C, Thomee R, Silbernagel KG, et al. Iontophoresis with or without dexamethazone in the treatment of acute Achilles tendon pain. *Scand J Med Sci Sports* 2003;13(6):376-82.

56. Dragoo JL, Braun HJ, Durham JL, et al. Comparison of the acute inflammatory response of two commercial platelet-rich plasma systems in healthy rabbit tendons. *AJSM* 2012.

57. Azevedo LB, Lambert MI, Vaughan CL, et al. Biomechanical variables associated with Achilles tendinopathy in runners. *Br J Sports Med* 2009;43(4):288-92.

58. Donoghue OA, Harrison AJ, Laxton P, et al. Lower limb kinematics of subjects with chronic achilles tendon injury during running. *Res Sports Med* 2008;16(1):23-38.

59. Moore KL. *Clinically oriented anatomy, 3^rd^ edn*. Baltimore: Williams and Wilkins, 1992.

60. Phillips LH, 2nd, Park TS. Electrophysiological mapping of the segmental innervation of the saphenous and sural nerves. *Muscle Nerve* 1993;16(8):827-31.

61. Bogduk N. *Clinical anatomy of the lumbar spine and sacrum*. 4th ed. London: Elsevier Churchill Livingstone, 2005.

62. Alfredson H, Cook J. A treatment algorithm for managing Achilles tendinopathy: new treatment options. *Br J Sports Med* 2007;41(4):211-6.

63. Han JS. Acupuncture analgesia: areas of consensus and controversy. *Pain* 2011;152(3 Suppl):S41-8.

64. Ellis A, Wiseman N, Boss K. *Fundamentals of Chinese Acupuncture*. Brooklyn, Massachusetts: Paradigm Publications, 1991.

65. Alfredson H, Pietila T, Jonsson P, et al. Heavy-load eccentric calf muscle training for the treatment of chronic Achilles tendinosis. *The American Journal of Sports Medicine* 1998;26(3):360-66.

66. Silbernagel KG, Thomee R, Thomee P, et al. Eccentric overload training for patients with chronic Achilles tendon pain--a randomised controlled study with reliability testing of the evaluation methods. *Scand J Med Sci Sports* 2001;11(4):197-206.

67. Robinson JM, Cook JL, Purdam C, et al. The VISA-A questionnaire: a valid and reliable index of the clinical severity of Achilles tendinopathy. *British journal of sports medicine* 2001;35(5):335-41.

68. Coombes BK, Bisset L, Vicenzino B. Efficacy and safety of corticosteroid injections and other injections for management of tendinopathy: a systematic review of randomised controlled trials. *Lancet* 2010;376(9754):1751-67.

69. Norkin CC, White DJ. *Measurement of joint motion a guide to goniometry*. 3rd ed. Philadelphia: Davis Company, 2003.

70. Kendall FP, McCreary EK, Provance PG, et al. *Muscles testing and function with posture and pain*. 5th ed. Baltimore: Lippincott Williams & Wilkins, 2005.

71. Elveru RA, Rothstein JM, Lamb RL. Goniometric reliability in a clinical setting. Subtalar and ankle joint measurements. *Phys Ther* 1988;68(5):672-7.
